# A Rare Dieulafoy Lesion in the Second Portion of the Duodenum: Successful Endoscopic Hemostasis

**DOI:** 10.7759/cureus.109987

**Published:** 2026-05-31

**Authors:** Intissar Lasfar, Oualid Hadadia, Khalid Gharbi, Rachid Akka

**Affiliations:** 1 Gastroenterology, Avicenne Military Hospital, Marrakech, MAR; 2 Gastroenterology and Hepatology, Moulay Hassan Hospital Guelmim, Guelmim, MAR; 3 Hepatogastroenterology, Avicenne Military Hospital, Marrakech, MAR

**Keywords:** dieulafoy's ulcer, duodenum, endoscopy, hemostasis, metal clips, upper gastrointestinal bleeding

## Abstract

Dieulafoy's ulcer is a rare but potentially fatal vascular malformation characterized by the presence of an abnormally large submucosal arteriole that erodes the overlying mucosa without an identifiable primary ulceration. Its duodenal location is exceptional. We report the case of a 76-year-old patient with multiple comorbidities, admitted to the emergency department of the Avicenne Military Hospital in Marrakech for melena that had been progressing for four days, accompanied by severe anemia (Hb 5.4 g/dL). An upper gastrointestinal endoscopy (EGD) revealed a Dieulafoy's lesion in the second part of the duodenum (D2), with spontaneous, diffuse bleeding and no visible ulceration. Endoscopic hemostasis was achieved by injection of adrenaline (6 cc) followed by the placement of two hemostatic metal clips, with a good clinical outcome.

## Introduction

Dieulafoy's ulcer, also called exulceratio simplex according to the historical terminology of the French surgeon Paul Georges Dieulafoy (1839-1911), is a rare but serious cause of upper gastrointestinal bleeding (UGIB) [1]. It accounts for approximately 1 to 2% of non-varicose UGIB cases [2]. The lesion consists of an abnormally large (1 to 3 mm, compared to the usual 0.1 mm) and tortuous submucosal arteriole protruding through a minimal mucosal erosion, without an adjacent perilesional ulcer [3]. Gastrointestinal involvement represents more than 70% of cases [3]. Extragastric locations, particularly in the duodenum, remain exceptional [4-6]. Fewer than forty cases of duodenal Dieulafoy's lesion have been reported in the world literature [7]. We report the case of an elderly patient with multiple comorbidities, treated at the Avicenne Military Hospital in Marrakech, who presented with a Dieulafoy lesion in D2, successfully treated by upper gastrointestinal endoscopy. The objective of this report is to describe the clinical and endoscopic characteristics of this rare condition, to discuss the therapeutic modalities, and to raise the issue of managing antithrombotic therapies in the context of active gastrointestinal bleeding [8].

## Case presentation

The patient was a 76-year-old man with multiple comorbidities: long-standing hypertension, a history of stroke without neurological sequelae, dyslipidemia, obstructive sleep apnea syndrome, type 2 diabetes requiring insulin since 2021, and end-stage renal disease with a baseline creatinine level of 40 mg/L. An arteriovenous fistula (AVF) of the left anatomical snuffbox had been created in January 2024 for hemodialysis access. The patient reported no known drug allergies. His usual treatment included: potassium chloride 600 mg, two tablets/day, urapidil 30 mg, two tablets/day, furosemide 40 mg/day, zolpidem 7.5 mg at bedtime, low-dose acetylsalicylic acid 75 mg/day, moxonidine 0.2 mg/day, ramipril 5 mg/day, repaglinide 1 mg before each main meal, amlodipine-enalapril 5 mg/20 mg/day, cholecalciferol 100,000 IU/quarter, and epoetin beta 40 µg/week subcutaneously for anemia of chronic renal failure.

The patient presented to the emergency department, referred by his primary care physician, with active melena that had been progressing for four days, associated with profound asthenia. His primary care physician had previously ordered blood tests, which revealed microcytic hypochromic anemia with a hemoglobin (Hb) level of 7 g/dL, prompting his emergency referral. Upon arrival at the emergency department, the patient was conscious and oriented (Glasgow Coma Scale 15/15), hemodynamically stable with a blood pressure of 130/75 mmHg, a heart rate of 88 bpm, an oxygen saturation of 97% on room air, a respiratory rate of 16 breaths/minute, and a temperature of 37.1°C. Physical examination revealed a pale, asthenic patient with a soft abdomen that was slightly tender in the epigastric region to palpation, without guarding or rigidity. Cardiovascular auscultation revealed no additional murmur; the left anatomical snuffbox artery was pulsatile with a palpable thrill. Neurological examination revealed no focal deficits residual from the previous stroke. Pulmonary auscultation was unremarkable. Digital rectal examination confirmed the presence of black stools (melena).

The blood tests performed in the emergency department showed microcytic hypochromic anemia with a hemoglobin level of 5.4 g/dL, a mean corpuscular volume (MCV) of 68 fL (microcytosis), a mean corpuscular hemoglobin (MCH) of 23 pg, a white blood cell count of 8,200/mm³, and a platelet count of 215,000/mm³; iron studies showed low, ferritin at 8 ng/mL (confirmed iron deficiency), low serum iron at 8 µmol/L, and a decreased transferrin saturation at 15%; coagulation studies showed no significant coagulopathy: prothrombin time (PT) of 78%, activated partial thromboplastin time (APTT) 1.15 times the control, and fibrinogen at 3.1 g/L; no electrolyte imbalances with sodium at 138 mmol/L, potassium at 4.8 mmol/L, and normal chloride levels at 98 mmol/L; creatinine at 68 mg/L (above the baseline value of 40 mg/L, consistent with slight degradation in the context of hemorrhage) and blood urea nitrogen (BUN) at 18 mmol/L; liver function tests within normal limits: aspartate amino transferase (AST) at 18 IU/L, alanine amino transferase (ALT) at 26 IU/L, total bilirubin at 12 µmol/L, gamma-glutamyl transferase (GGT) at 24 UI/L, and alkaline phosphatase (ALP) at 52 IU/L (Table 1).

**Table 1:** Laboratory results on admission to the emergency department

Parameter	Patient value	Normal range
Hemoglobin (Hb)	5.4 g/dL	13–17 g/dL
Mean corpuscular volume (MCV)	68 fL (microcytosis)	80–100 fL
Mean corpuscular Hb (MCH)	23 pg (hypochromia)	27–33 pg
White blood cells	8,200/mm³	4,000–10,000/mm³
Platelets	215,000/mm³	150,000–400,000/mm³
Ferritin	8 ng/mL	20–300 ng/mL
Serum iron	8 µmol/L	11–29 µmol/L
Transferrin saturation	15%	20–45%
Prothrombin time (PT)	78%	70–100%
Activated partial thromboplastin time	1.15 × control	< 1.2 × control
Fibrinogen	3.1 g/L	2–4 g/L
Serum sodium	138 mmol/L	135–145 mmol/L
Serum chloride	98 mmol/L	96–106 mmol/L
Serum potassium	4.8 mmol/L	3.5–5.0 mmol/L
Creatinine	68 mg/L (baseline: 40mg/L)	5–14 mg/L
Blood urea nitrogen (BUN)	18 mmol/L	2.5–7.5 mmol/L
Aspartate aminotransferase (AST)	18 IU/L	<40 IU/L
Alanine aminotransferase (ALT)	26 IU/L	<40 IU/L
Total bilirubin	12 µmol/L	<17 µmol/L
Gamma-glutamyl transferase (GGT)	24 IU/L	<50 IU/L
Alkaline phosphatase (ALP)	52 IU/L	30–125 IU/L

Initial management in the emergency department consisted of inserting a peripheral intravenous line. An intravenous proton pump inhibitor (PPI) was started upon admission (esomeprazole 80 mg bolus followed by 8 mg/hour), in accordance with current guidelines [9]. Medications potentially implicated in the bleeding were temporarily discontinued: Kardegic®, Eupressyl®, and ramipril. Given the severe anemia (hemoglobin 5.4 g/dL), two units of Rh- and blood-matched packed red blood cells were transfused, resulting in a post-transfusion hemoglobin level of 8.2 g/dL. Aspirin was discontinued in the acute phase according to the 2022 American College of Gastroenterology-Canadian Association of Gastroenterology (ACG/CAG) guidelines [10], with a subsequent reassessment of the benefit-risk ratio in consultation with the neurology and cardiology teams.

An emergency upper gastrointestinal endoscopy (EGD) was performed under local anesthesia with pharyngeal lidocaine spray, with the patient in the left lateral decubitus position. The esophagus appeared normal; a 3 cm sliding hiatal hernia was noted. The stomach showed no hemorrhagic lesions; only bilious stasis fluid was found and aspirated. The antrum and duodenal bulb were unremarkable.

**Figure 1:**
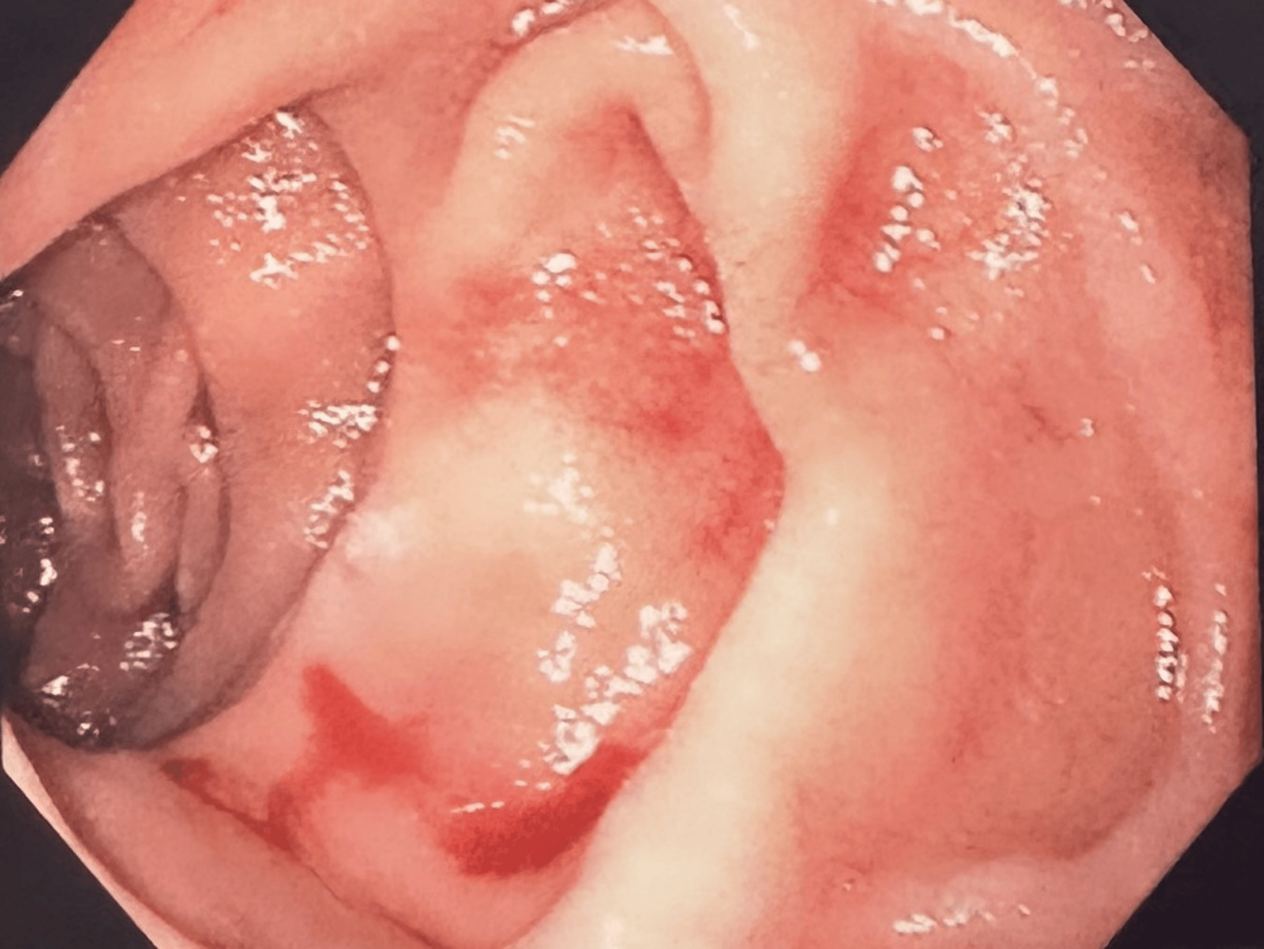
Spontaneous, diffuse bleeding at the level of D2

Upon passage into the second part of the duodenum (D2), in the post-bulbar position, spontaneous, bright red bleeding was identified, emanating from a punctate mucosal point, without any visible perilesional ulceration, granulation tissue, or overlying mass. The appearance was highly characteristic of a duodenal Dieulafoy's ulcer (Figure 1).

**Figure 2:**
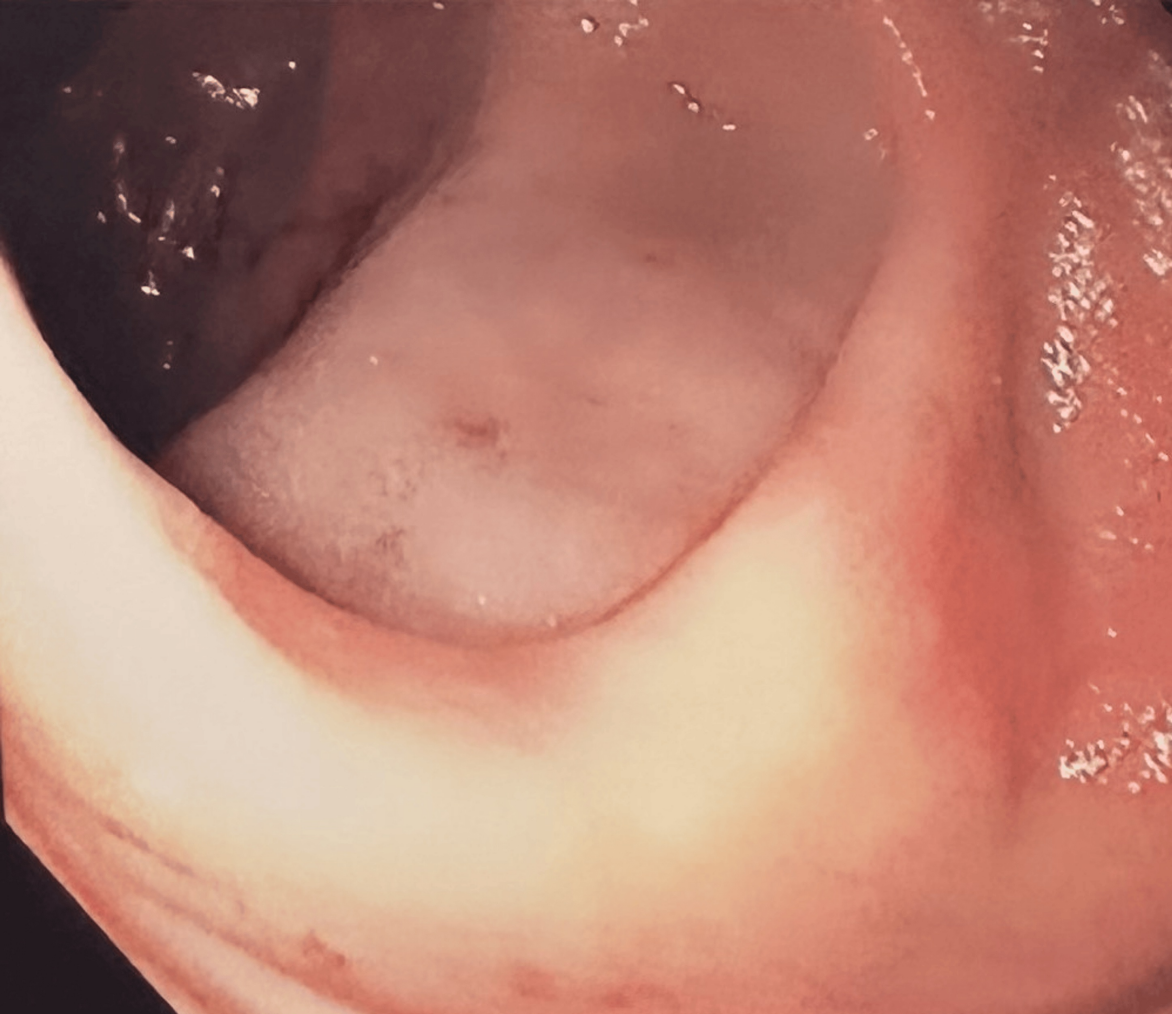
Hemostasis after adrenaline injection

The therapeutic endoscopic procedure consisted of a local injection of 6 cc of adrenaline diluted 1/10,000 to the immediate periphery of the lesion, achieving initial hemostasis through vasoconstriction (Figure 2).

**Figure 3:**
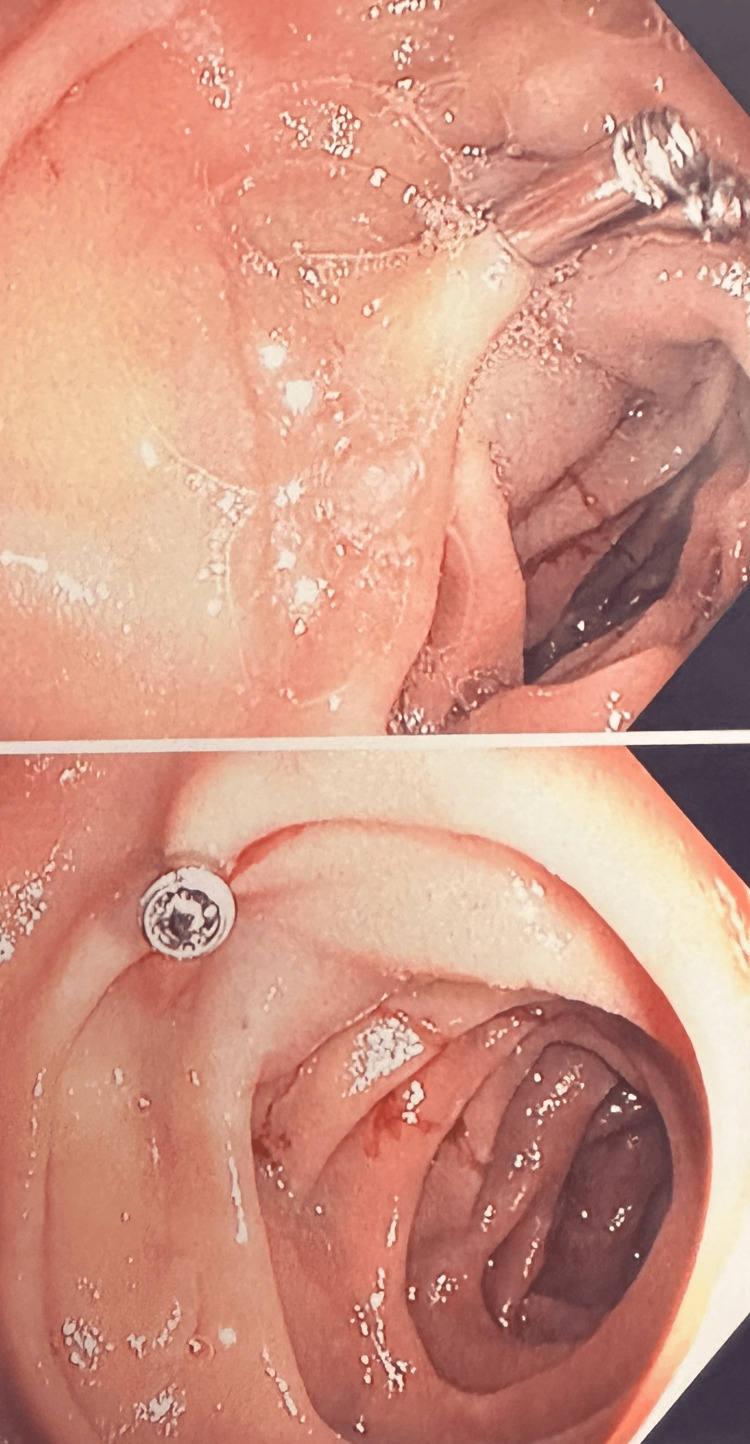
View after placement of the two hemostatic metal clips

Due to the known risk of rebleeding with injection alone, two hemostatic metal clips were placed at the site of the lesion to ensure sustained mechanical compression of the submucosal vessel (Figure 3). At the end of the procedure, hemostasis was achieved and confirmed.

Following the endoscopic procedure, the patient was hospitalized in a care unit with close clinical and laboratory monitoring. A continuous intravenous proton pump inhibitor (PPI) was administered for 72 hours, then switched to oral administration (esomeprazole 40 mg/day). A *Helicobacter pylori* serology test was routinely performed and came back negative. The clinical course was favorable: no recurrent bleeding, and progressive normalization of stool color by day 5. A complete blood count (CBC) on day 5 showed a stable hemoglobin level of 8.6 g/dL. The patient was discharged on day 8, continuing oral PPIs for six weeks, with close follow-up in a hepatogastroenterology consultation.

The resumption of aspirin (Kardegic® 75 mg) was the subject of a multidisciplinary discussion. In accordance with the ACG/CAG 2022 recommendations [10] and Asian guidelines [11], given the history of stroke (secondary prevention) and the absence of hemorrhagic recurrence, the resumption of low-dose acetylsalicylic acid was decided on day 5 post-endoscopy, under PPI coverage, after neurological consultation.

## Discussion

Dieulafoy's ulcer is characterized by a persistent submucosal arteriole (1 to 3 mm in diameter, or 10-15 times the normal diameter), which creates abnormally high pulsatile pressure on the mucosa, potentially leading to punctate erosion and spurting bleeding [2,3]. Men over 70 years of age with hypertension and taking antiplatelet agents represent the predominant epidemiological profile [4]. Our patient had several risk factors: hypertension, end-stage renal disease, diabetes, and chronic aspirin use.

Upper gastric location accounts for at least 71% of cases [5]. Duodenal location accounts for approximately 15% of cases [6]. Lesions in the second part of the duodenum (D2), as in our case, make endoscopic access more difficult and partly explain delayed diagnoses [7,8].

The clinical presentation is that of sudden, massive, recurrent gastrointestinal bleeding in a patient with no history of ulcers [2]. The first endoscopy is non-diagnostic in up to 51% of cases due to the small size of the lesion and its difficult location [3]. In cases of doubt, the literature recommends a second early endoscopy, endoscopic ultrasound, or even selective mesenteric angiography [9].

In our case, an EGD allowed for a definitive diagnosis during a single examination, thanks to the visualization of spontaneous, diffuse bleeding from a pinpoint location without an ulcer in the second part of the duodenum (D2) [6,7]. This underscores the importance of a systematic and thorough duodenal exploration during any endoscopy for upper gastrointestinal bleeding of undetermined origin.

Therapeutic endoscopy is the first-line treatment for Dieulafoy lesions, with a primary hemostasis rate exceeding 90% [12]. Current consensus recommends combined therapy (adrenaline injection + clips) to reduce the risk of recurrence [3,12]. Clip placement is the gold standard for extragastric duodenal lesions due to the increased risk of perforation associated with thermal techniques on a thin duodenal wall [9].

Initiation of continuous intravenous PPI therapy following endoscopic hemostasis of a non-varicose hiatal hernia is a well-established practice [13]. The protocol of esomeprazole 80 mg as a bolus followed by 8 mg/hour for 72 hours, followed by oral administration, is recommended in cases of lesions at high risk of recurrence [13], a category to which Dieulafoy's lesion belongs.

The 2022 ACG/CAG guidelines specify that in patients taking aspirin for secondary cardiovascular prevention, discontinuation exposes them to a major thrombotic risk [10]. Asian guidelines recommend resuming the antiplatelet agent on the first to third day post-hemostasis [11]. In our case, resuming treatment on day 5 with PPI coverage, after neurological consultation, illustrates the importance of a multidisciplinary approach for patients in secondary prevention of cerebral ischemic events.

In cases of failure of endoscopic hemostasis, selective arterial embolization and hemostatic surgery remain alternatives [14,15]. In our case, the effectiveness of the endoscopic treatment rendered these alternatives unnecessary.

## Conclusions

Dieulafoy's lesion of the second part of the duodenum is a rare and potentially fatal condition if overlooked, and should be included in the differential diagnosis of any upper gastrointestinal bleeding of initially undetermined origin. This case, managed at the Avicenne Military Hospital in Marrakech, illustrates several key points: the necessity of a complete endoscopic exploration down to the second part of the duodenum (D2) in any case of unexplained upper gastrointestinal bleeding; the efficacy and safety of combined endoscopic treatment (injection of adrenaline + two hemostatic metal clips) in this location, with primary hemostasis achieved in a single procedure; and finally, the importance of a multidisciplinary approach for managing antithrombotic therapies in the acute phase, particularly for the secondary prevention of cerebral ischemic events. The dissemination of documented clinical cases, accompanied by high-quality endoscopic images, helps raise awareness among practitioners of this rare pathology and improve its early recognition.
